# Regulation of DNA repair gene expression by PRMT5

**DOI:** 10.17912/micropub.biology.001631

**Published:** 2025-07-14

**Authors:** Hunter Bliss, Renee A Bouley, Ruben C Petreaca

**Affiliations:** 1 Biology, The Ohio State University; 2 The Ohio State University

## Abstract

PRMT5 is a member of a class of enzymes called protein arginine methyltransferases (PRMTs) that play a role in maintaining genomic stability through post-translational modification of components of the TIP60 chromatin remodeling super complex. TIP60 is required primarily for chromatin remodeling at DNA double-strand breaks. Mutations in either TIP60 or PRMT5 affect repair by homologous recombination. A recent study has shown that in leukemia and lymphoma, PRMT5 also controls mRNA expression of TIP60 and other DNA repair genes by regulating alternative splicing. This analysis utilizes publicly available data from the Catalogue of Somatic Mutations in Cancer (COSMIC) to interrogate how PRMT5 expression correlates with expression levels of other key DNA repair genes: KAT5 (TIP60), H2A (H2AFX), TP53, TP53BP1, and RAD51 in all cancers. We find that indeed an increase in PRMT5 expression levels correlates with an increase in KAT5 levels. A weak correlation was also observed between PRMT5 and TP53, TP53BP1, and RAD51. These findings extend previous PRMT5 roles in controlling gene expression to all cancer types and further highlight the role of this enzyme not only in post-translational modification but also regulation of gene expression.

**Figure 1. Correlation between PRMT5 expression and DNA repair gene expression in cancer samples:**
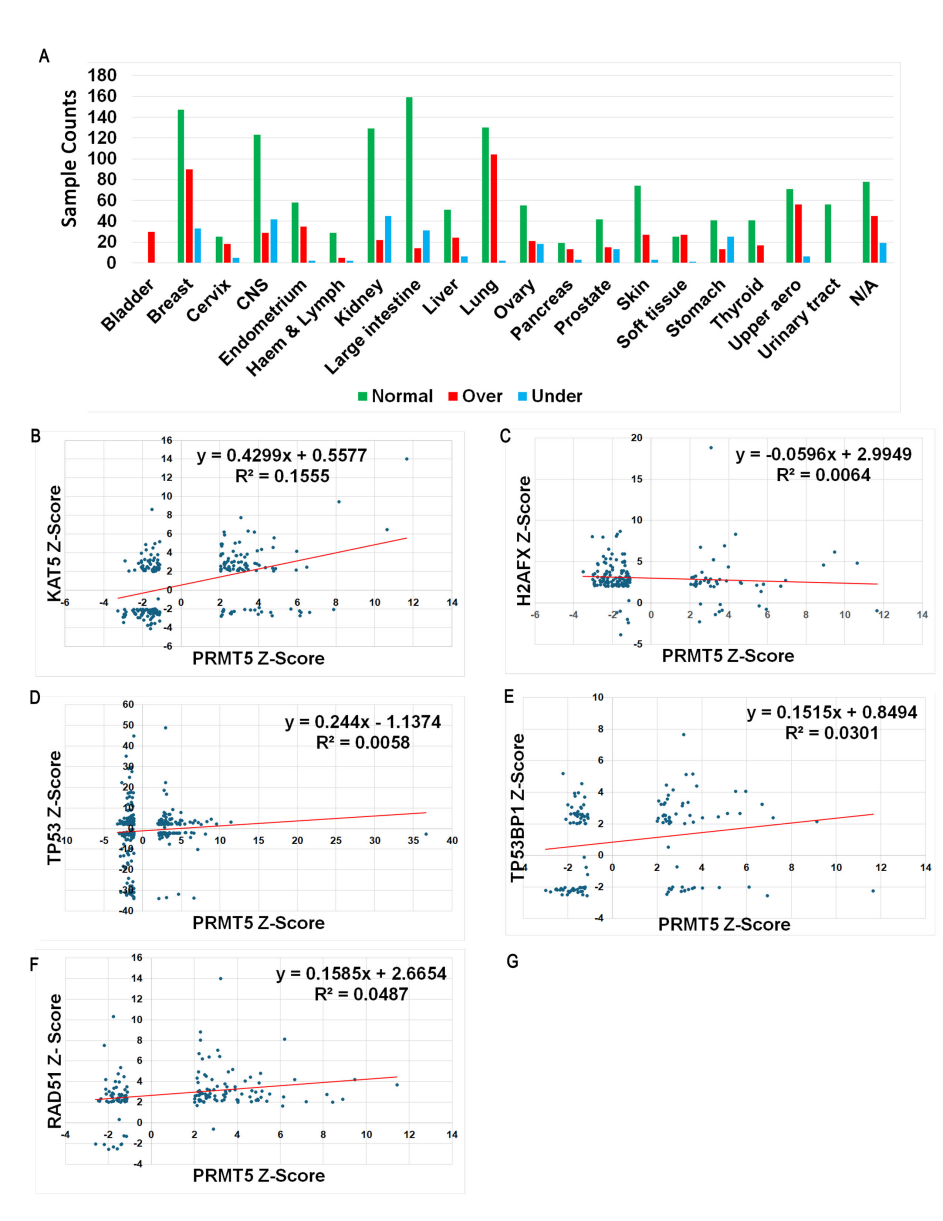
**(A)** The distribution of PRMT5 expression levels across different cancer types. Z-scores for TCGA samples for the indicated genes were separated into three categories: normal expression (green: Z-scores between -2 and +2), over-expressed (red: Z-scores over +2), and under-expressed (blue: Z-scores under -2). The Y-axis indicates counts of samples in each category. Please see text description for more details on interpretation of Z-scores. **(B-F)** Scatter plots showing the correlation between PRMT5 and the DNA repair genes KAT5 (TIP60), H2A (H2AFX), TP53, TP53BP1, and RAD51. PRMT5 expression is indicated on the x-axis, while the expression of the other repair genes is indicated on the y-axis. The data points represent individual samples, and the red line indicates the linear regression trend.

## Description

PRMT5, a type II protein arginine methyltransferase with pleiotropic functions, including regulating the function of chromatin remodeling enzymes involved in the repair of DNA double-strand break repair (DSB) by homologous recombination (Bedford 2007, Clarke, Sanchez-Bailon et al. 2017, Motolani, Martin et al. 2021). PRMT5 mutations are endemic in cancer cells, suggesting that its function is required for the maintenance of genomic stability (Rasheed, Bouley et al. 2023, Al-Marrawi, Petreaca et al. 2025). Further, PRMT5 small-molecule inhibitors are already being developed and even deployed as therapeutic targets, highlighting the importance of this gene in cellular transformation and immortalization (Gao, Yang et al. 2023, Zheng, Li et al. 2023, Jiao, Huang et al. 2024).

The primary function of PRMT5 in DSB repair is to methylate RUVBL1 (Clarke, Sanchez-Bailon et al. 2017), a component of the TIP60 histone acetyltransferase super complex (Chen, Wang et al. 2024, Yang, Mameri et al. 2024). KAT5, the acetyltransferase enzymatic subunit of TIP60, is conserved from yeast to humans, and we and others have shown that mutations directly affect repair of DSBs by homologous recombination (Sun, Jiang et al. 2010, Xu and Price 2011, Li, Petreaca et al. 2021, Li, Petreaca et al. 2024). A recent study showed that PRMT5 also regulates the alternative splicing and expression of the KAT5 (TIP60) enzyme in fetal and adult bone marrow cell lines, and this function is also required for DSB repair (Hamard, Santiago et al. 2018). This suggests that PRMT5 functions in DSB repair are more complex and extend from the regulation of transcription and modulation of protein function by post-translational modifications. In this report, we queried the Catalogue of Somatic Mutations in Cancer (COSMIC) database (Sondka, Dhir et al. 2024) to understand how changes in PRMT5 mRNA expression levels affect the expression of other DNA damage repair genes. We focused on interrogating the mRNA expression relationship between PRMT5 and the DSB repair genes, KAT5, H2AFX, TP53, TP53BP1, and RAD51, which the previous publication has shown are controlled by PRMT5. The goal was to understand whether PRMT5 regulation of gene expression extends to other cancers.

COSMIC reports expression levels for TCGA data. PRMT5 expression data were sorted into three categories: normally expressed (green), over-expressed (red), and under-expressed (blue). COSMIC presents expression data normalized to control samples as Z-scores. Z-scores are interpreted as under-expressed if they are below -2, normally expressed if they are between -2 and +2, and over-expressed if they are above +2 (Cheadle, Vawter et al. 2003). The distribution of PRMT5 expression levels indicates that most cancer types are characterized by normal PRMT5 expression levels ( **Fig.1A** ). Interestingly, bladder cancers only had data showing over-expression of PRMT5, while cancers of the urinary tract had only normal expression of PRMT5, although this may be due to a small sample size.

In **Figures 1B-1F** , by correlating the Z-score of PRMT5 expression with the Z-score of other DNA repair genes from the same sample via linear regression, the following observations were made: a moderately positive correlation (R ^2^ = 0.1555) between PRMT5 and KAT5; a weak negative correlation (R ^2^ = 0.0064) between PRMT5 and H2A (H2AFX); a weak positive correlation (R ^2^ = 0.0059) between PRMT5 and TP53; a weak positive correlation (R ^2^ = 0.0301) between PRMT5 and TP53BP1; and a weak positive correlation (R ^2^ = 0.0487) between PRMT5 and RAD51. This brief analysis indeed suggests that an increase in PRMT5 expression correlates with an increase in KAT5 expression and, to a lesser degree, TP53, TP53BP1, and RAD51. Expression of PRMT5 does not appear to affect H2AFX expression. These data agree with the previous work showing that PRMT5 controls primarily KAT5 splicing as well as other associated histone-modifying genes (Hamard, Santiago et al. 2018). Additionally, it suggests that this function extends to all cancer types, and depletion or inhibition of PRMT5 could be used as a therapeutic cancer treatment target.

## Methods

***Data Accession*** . A data set consisting of PRMT5 expression data was downloaded from the COSMIC database. This Excel file consisted of three individual data sheets, each corresponding to an expression level of PRMT5: normal expression, over-expression, and under-expression. For each sample within the dataset, the corresponding z-score for the other DNA repair genes was input by manually searching the sample IDs within the COSMIC database. Demographics such as age, sex, and cancer type for each sample were also manually input by searching the sample IDs within COSMIC.

***Data Analysis and Visualization*** . To investigate the gene-gene expression relationships between PRMT5 and other DNA repair genes, scatter plots were utilized for each relationship. The data points for plotted for each sample with the PRMT5 z-score on the x-axis and the z-score of the other genes on the y-axis. For some samples, there was data for multiple genes, while others had a data deficit, resulting in partial data points across the other scatter plots. The resulting scatter plots were analyzed via Excel to identify that the strength of the gene-gene expression relationship. A simple linear regression analysis was performed to quantify the strength of these relationships with an R ^2^ value. Values with a greater R ^2^ value indicates a stronger gene-gene interaction.
